# BPTF promotes tumor growth and predicts poor prognosis in lung adenocarcinomas

**DOI:** 10.18632/oncotarget.5302

**Published:** 2015-09-21

**Authors:** Meng Dai, Jian-Jun Lu, Wei Guo, Wendan Yu, Qimin Wang, Ranran Tang, Zhipeng Tang, Yao Xiao, Zhenglin Li, Wei Sun, Xiuna Sun, Yu Qin, Wenlin Huang, Wu-guo Deng, Taihua Wu

**Affiliations:** ^1^ The First Affiliated Hospital & Institute of Cancer Stem Cell, Dalian Central Hospital, Dalian Medical University, Dalian, China; ^2^ Sun Yat-Sen University Cancer Center, State Key Laboratory of Oncology in South China, Collaborative Innovation Center of Cancer Medicine, Guangzhou, China; ^3^ Department of Thoracic Surgery, The First Affiliated Hospital, Sun Yat-sen University Cancer Center, Guangzhou, China; ^4^ The Second Affiliated Hospital, Dalian Medical University, Dalian, China; ^5^ State Key Laboratory of Targeted Drug for Tumors of Guangdong Province, Guangzhou Double Bioproduct Inc., Guangzhou, China

**Keywords:** BPTF, lung cancer, tumor growth, prognosis

## Abstract

BPTF, a subunit of NURF, is well known to be involved in the development of eukaryotic cell, but little is known about its roles in cancers, especially in non-small-cell lung cancer (NSCLC). Here we showed that BPTF was specifically overexpressed in NSCLC cell lines and lung adenocarcinoma tissues. Knockdown of BPTF by siRNA significantly inhibited cell proliferation, induced cell apoptosis and arrested cell cycle progress from G1 to S phase. We also found that BPTF knockdown downregulated the expression of the phosphorylated Erk1/2, PI3K and Akt proteins and induced the cleavage of caspase-8, caspase-7 and PARP proteins, thereby inhibiting the MAPK and PI3K/AKT signaling and activating apoptotic pathway. BPTF knockdown by siRNA also upregulated the cell cycle inhibitors such as p21 and p18 but inhibited the expression of cyclin D, phospho-Rb and phospho-cdc2 in lung cancer cells. Moreover, BPTF knockdown by its specific shRNA inhibited lung cancer growth *in vivo* in the xenografts of A549 cells accompanied by the suppression of VEGF, p-Erk and p-Akt expression. Immunohistochemical assay for tumor tissue microarrays of lung tumor tissues showed that BPTF overexpression predicted a poor prognosis in the patients with lung adenocarcinomas. Therefore, our data indicate that BPTF plays an essential role in cell growth and survival by targeting multiply signaling pathways in human lung cancers.

## INTRODUCTION

Lung cancer has become the first tumor in morbidity and mortality among males, and has been the leading cause of cancer death among females in more developed countries [1]. Although there is a rapid development in the treatment by targeted therapies, the five-year survival rate is still quite low, less than 16% all over the world [1, 2]. One of the main reasons for this is that poorly is known about the biology of lung cancer, so that we cannot find effective ways to cure it. Therefore, fully understanding the essence of the cell biology and molecular biology of lung cancer is a key way to conquer it.

It is well known that chromatin remodeling is closely associated with tumors [3, 4]. It is a basic procedure for eukaryotic gene expression [5]. DNA is tightly packaged in nucleus. Such condensed structure inhibits transcription factors binding to promoter regions, which leads to restrain of gene expression. Numbers of evidence prove chromatin remodeling is essential for cell growth and division. Mutations in such chromatin remodelers and deregulated covalent histone modifications potentially make the cell growth out of control and then initiate cancers [6, 7]. For example, inactivating mutations in SMARCB1, a component of the human SWI/SNF remodeling complex, can lead to rhabdoid tumors [8]. Mutations in histone acetyl transferases (HAT) p300 are found in lung cancer, pancreatic, colorectal, breast cancer and so on [9–11].

Chromatin remodeling is carried out mainly by covalent histone-modifying complexes and ATP-dependent chromatin remodeling complexes. The frontier includes histone acetyltransferases (HATs), deacetylases, methyltransferases and kinases; the later contains SWI/SNF, ISWI, NuRD/Mi-2/CHD, INO80 and SWR1 [12, 13]. NURF (nucleosome remodeling factor) is one of ISWI super family members. It was originally characterized in Drosophila, which worked in nucleosome assembly and spacing *in vitro* and regulated transcription of several hundreds of Drosophila genes *in vivo*, such as vital genes for fly development [14, 15]. NURF contains 4 subunits in Drosophila, whereas it includes 3 subunits in human. Both of the NURFs are highly homologous, which strongly indicates that it has been conserved through evolution. Also it indicates that the factor has a basic and important effect on life [12, 16].

BPTF, called bromodomain PHD-finger transcription factor, is the largest unit of NURF. In the previous reports, most information about BPTF focused on the regulation of genes essential for the development of key tissues and chromatin remodeling [17–19]. BPTF was also reported to be able to regulate Erk expression in melenoma [26]. Although it is an indisputable fact that transcription factors are intimately associated with cancers, little is known about the roles of BPTF in cancers. Some authors reported that *bptf* gene was mutated in bladder cancer, and the mutated *bptf* gene could promote lung pre-malignant, which also showed knocking down *bptf* led to a dramatic reduction in colony formation [20, 21]. In addition, some authors reported that BPTF indicated a negative prognosis in patients with hepatocellular carcinoma [11]. All these researches indicate that BPTF may be a cancer-promoting protein. However, we know little about its biological behavior and its further molecular mechanisms in cancers, especially in NSCLC.

In this study, we examined the effects of BPTF on lung cancer cell proliferation, apoptosis and cell cycle, and further identified the underlying molecular mechanisms *in vitro* and *in vivo*. We also assessed whether BPTF expression was correlated with the overall survival in lung adenocarcinoma. Our findings therefore provide a new understanding about NSCLC tumorigenesis, and maybe support a fresh potential choice of therapeutic target for lung cancers.

## RESULTS

### BPTF was highly expressed in NSCLC cell lines and tumor tissues

**Figure 1:**
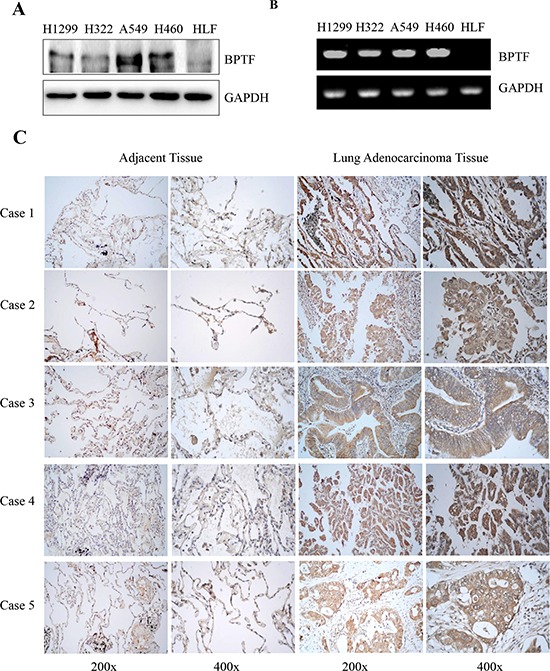
BPTF was overexpressed in NSCLC cell lines and lung adenocarcinoma tissues **A.** The expression of BPTF in human normal lung cell HLF and various NSCLC cell lines was analyzed by Western blot. **B.** The expression of BPTF in human normal lung cell HLF and various NSCLC cell lines was analyzed by RT-PCR. **C.** The expression of BPTF in human lung adenocarcinoma and their adjacent non-malignant lung tissues from 5 different patients with lung cancers (Case 1, 2, 3, 4 and 5) was detected by immunohistochemical staining (magnification, 200x and 400x).

We selected the human normal lung cell line HLF and 4 NSCLC cell lines (A549, NCI-H460, H322 and H1299) for our study. We checked the BPTF expression in NSCLC cell lines by Western Blot and RT-PCR (Figure 1A, 1B). Then we detected the percentage of positive BPTF expression in lung adenocarcinoma tissue by immunohistochemistry assay on a tissue microarray of 75 cases with cancer tissues and adjacent nonmalignant lung tissues (Figure 1C). Immunohistochemical staining showed that lung tumor tissues had much higher expression of BPTF than their adjacent non-cancer tissues. Five representative cases (Case 1 to 5) were respectively from 5 different patients (Figure 1C). Among 75 patients’ tumor tissue samples tested, about 53 (70.7%) patients showed high expression of BPTF. These results indicated BPTF might be a potential biomarker for human lung cancer.

### Knockdown of BPTF inhibited NSCLC cell growth and survival *in vitro*

**Figure 2:**
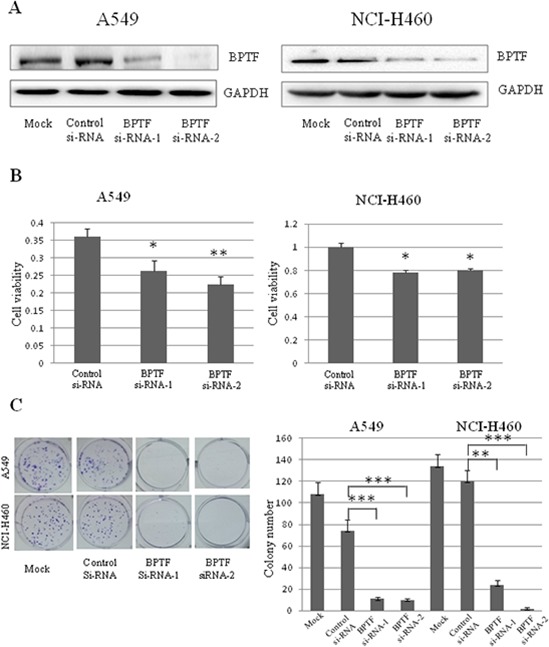
Knockdown of BPTF inhibited proliferation of NSCLC cells **A.** The expression of BPTF in A549 and NCI-H460 cell lines were analyzed by Western blot after transfected with BPTF siRNA. Mock, non treatment; control siRNA, non specific siRNA; BPTF siRNA-1 and BPTF siRNA-2, BPTF specific siRNA. **B.** Cell viability of A549 and NCI-H460 cells was measured by MTT assay. The mean and SD value got from 3 independent experiments are marked (*P < 0.05; **P < 0.01). **C.** Colonies (>50 μm) were counted 10–12 days in A549 and NCI-H460 cells after transfected by siRNA. Each bar represents the mean colony number and SD of 3 wells (*P < 0.05; **P < 0.01).

To study the biological function of BPTF protein, we selected NSCLC cell lines (A549, H460, H322) as the cell models which highly expressed BPTF. Since the BPTF gene is quite large, it is hard to acquire the plasmid with the complete sequence of BPTF. Therefore, we applied RNA interference technology to study the function of BPTF in NSCLC cells. The non-specific small interfering RNA (siRNA) and BPTF-siRNAs were transfected into A549 and NCI-H460 cells. 48 hours after treatment, BPTF was markedly down regulated in these 2 cell lines (Figure 2A). At the same time, knockdown of BPTF significantly inhibited cell viability by MTT assay (Figure 2B), and clone formation was also suppressed compared with the control group transfected with the non-specific siRNA (Figure 2C). These results showed that BPTF played a crucial role in cell proliferation of NSCLC cells.

### Knockdown of BPTF inactivated MAPK and PI3K/AKT signaling pathways

**Figure 3:**
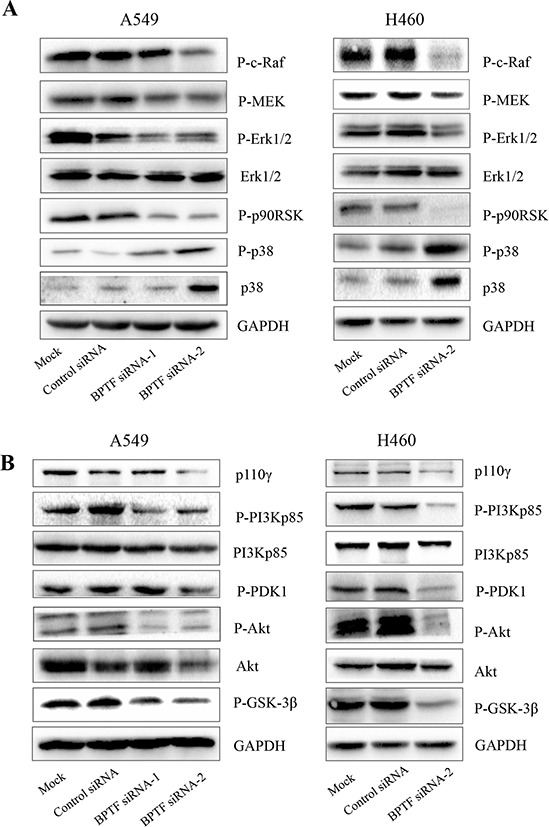
Knockdown of BPTF suppressed MAPK and PI3K-AKT signaling pathways **A.** The key protein in MAPK pathways were detected by immunoblot in A549 and NCI-H460 cells 3 days after transfected by siRNA. **B.** The proteins in PI3K-AKT pathway in A549 and NCI-H460 were analyzed by Western blot 3 days after siRNA transfection.

To deliberate the molecular mechanism by which BPTF regulated cell proliferation in NSCLC cells, we detected a number of important proteins related with tumorigenesis by immunoblot (Figure 3). We found that when BPTF was knocked down, phospho-c-Raf, phospho-MEK1/2, phospho-Erk1/2 and phospho-p90RSK were decreased in the MAPK pathway, while phospho-p38 and p38 were increased. In the PI3K/AKT pathway, phospho-p85, p110γ, phospho-PDK1, phospho-Akt and phospho-GSK-3β were also reduced. These results indicate that BPTF knockdown-mediated inhibition of cell proliferation may be associated with the inhibition of the MAPK and PI3K/Akt pathways in NSCLC cell lines.

### BPTF knockdown induced cell apoptosis

**Figure 4:**
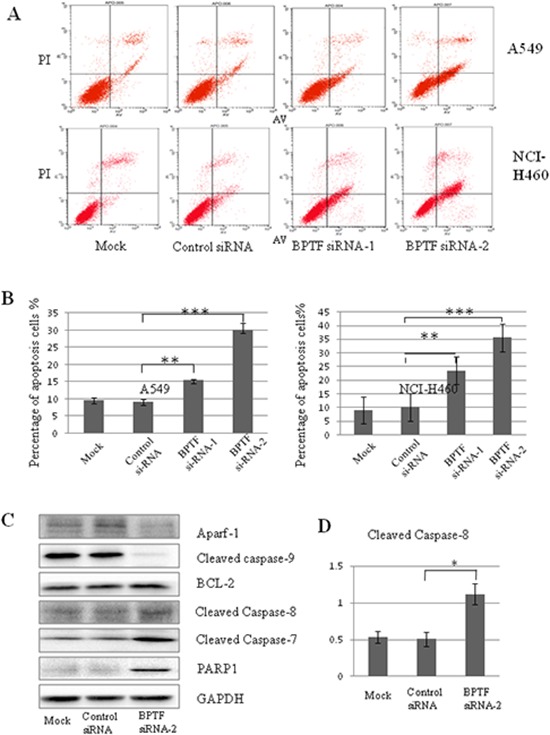
Knockdown of BPTF activated apoptosis by caspase-dependent pathway **A.** A549 and NCI-H460 cells transfected with siRNA for 3 days were analyzed by FACS using an Annexin V-FITC/PI-staining kit. **B.** Apoptosis was calculated in terms of the FITC-positive in cells. Each bar represents the mean and SD value of 3 experiments (**P < 0.01; ***P < 0.001). **C.** NCI-H460 cells with knockdown of BPTF were analyzed by Western blot with antibodies of Apaf-1, cleaved caspase-9, BCL-2, cleaved caspase-8, caspase-7 and PARP1. **D.** The quantitative analysis for the cleaved caspase-8.

To investigate the effect of BPTF on cell apoptosis, we performed Annexin V-PI staining-based FACS analysis. Knockdown of BPTF led to the increase of approximately 15%–20% of apoptotic cells compared with the control group in A549 and NCI-H460 cell lines (Figure 4A and 4B). To confirm this, we also analyzed the apoptosis-related molecules by Western blot. As shown in Figure 4C and 4D, knockdown of BPTF effectively increased the levels of cleaved caspase-8, cleaved caspase-7 and cleaved PARP1, but decreased the levels of Apaf-1, cleaved caspase-9 in NCI-H460 cells. These results indicate that BPTF plays an important role in the regulation of apoptosis in lung cancer cells.

### BPTF knockdown inhibited cell cycle progress from G1 to S phase

**Figure 5:**
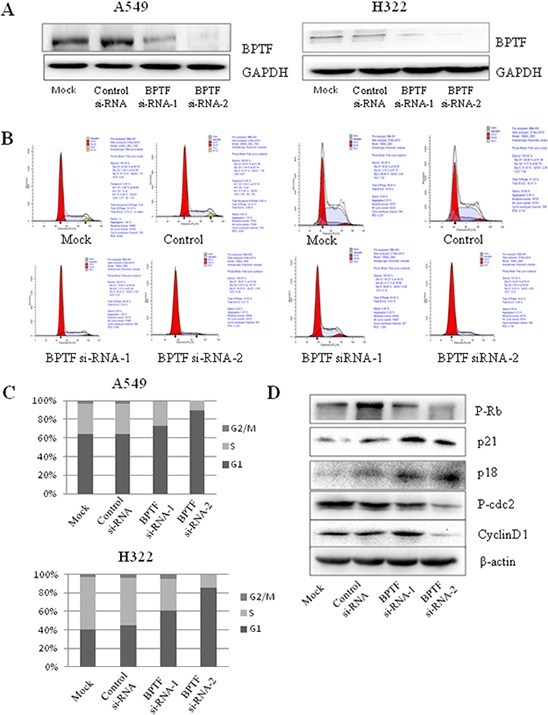
Knockdown of BPTF inhibited cell cycle **A.** BPTF was analyzed by Western blot in A549 and H322 cell lines transfected with BPTF siRNA for 3 days. **B.** Cell cycle of A549 and H322 cells with knockdown of BPTF was tested by FACS. **C.** Cell cycle was analyzed in terms of peaks of G1, S and G2/M. Each bar represents the SD from 3 independent experiments. **D.** Key proteins associated with cell cycle were detected by Western blot.

We next assessed the effect of BPTF on cell cycle progress by a PI staining-based FACS analysis in A549 and H322 cell lines transfected with BPTF siRNA. As shown in Figure 5B and 5C, knockdown of BPTF resulted in more staining of cells in the G1 phrase but less cell staining in the S phrase by comparison with the nonspecific-siRNA group. Also, we detected the relevant vital proteins involved in cell cycle regulation from G1 to S phrase and cell cycle check points by Western blot (Figure 5D). We found that knockdown of BPTF inhibited the expression of cyclin D1 and phospho-Rb; whereas p21 and p18 were enhanced by BPTF knockdown compared with the control groups.

### BPTF predicted a poor prognosis in lung adenocarcinomas

**Figure 6:**
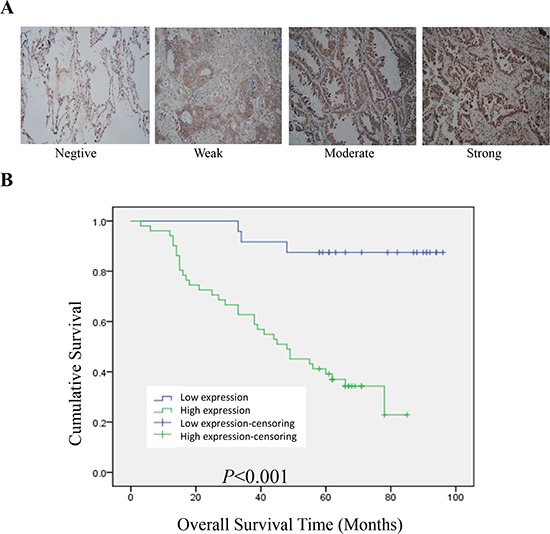
BPTF predicted a poor prognosis in lung adenocarcinoma **A.** Typical examples for negative, weak, moderate and strong BPTF expression in lung adenocarcinoma tissues (Magnification 200X). **B.** Overall survival of lung adenocarcinoma patients with high or low BPTF expression was analyzed by Kaplan-Meier analysis.

To further reveal the clinicopathologic significance of BPTF in lung adenocarcinomas, tissue microarray immunohistochemistry assay for 75 cases of lung adenocarcinomas was analyzed. The results showed that high expression of BPTF was associated with N (lymph node metastasis) and clinical staging factors (*P* < 0.05) according to Pearson chi-square test (Table 1A). N, clinical stage and BPTF expression were also associated with survival of the patients with lung adenocarcinomas based on Univariate analysis of Cox regression model (Table 1B). Furthermore, multivariate analysis revealed that the clinical stages and BPTF expression were the influencing factors to lifetime of lung adenocarcinomas (Table 1B). We also used Kaplan–Meier analysis to analyze the difference of overall survival between high and low BPTF expression (Figure 6B). The results showed that high expression of BPTF was negatively associated with the overall survival, indicating that BPTF predicted a poor prognosis in lung adenocarcinomas.

**Table 1A:** Association of BPTF with clinicopathological features in 75 cases with lung adenocarcinoma

	Total (*n* = 75)	BPTF high expression (*n* = 51)	BPTF low expression (*n* = 24)	*P*
Gender				
Male	39	23 (59%)	16 (41%)	0.081
Female	36	28 (77.8%)	8 (22.2%)	
Age				
<60	38	25 (65.8%)	13 (34.2%)	0.737
> = 60	36	25 (69.4%)	11 (30.6%)	
**T**				
T1 + T2	63	41 (65.1%)	22 (34.9%)	0.336
T3 + T4	12	10 (83.3%)	2 (16.7%)	
**N**				
N0 + N1	52	29 (55.8%)	23 (44.2%)	0.011
N2 + N3	12	12 (100%)	0 (0%)	
**Clinical Stage**				
Stage I	34	18 (52.9%)	16 (47.1%)	0.013
Stage II	12	6 (50.0%)	6 (50.0%)	
Stage III	16	15 (93.8%)	1 (6.3%)	
Stage IV	3	3 (100%)	0	

**Table 1B:** Cox regression model analysis of prognostic factors in 75 cases with lung adenocarcinoma

	RR	95%CI	Unfaourable/Favourable	*P*
**Univariate analysis**				
T	2.255	1.606–4.797	T1+T2/T3+T4	0.035
N	5.354	2.475–11.582	N0+N1/N2+N3	0.000
Clinical Stage	1.804	1.321–2.464	Stage I/II/III/IV	0.000
BPTF	8.543	2.587–28.217	High/Low	0.000
**Multivariate analysis**				
Clinical Stage	1.971	1.355–2.868	Stage I/II/III/IV	0.000
BPTF	8.034	2.244–28.762	High/Low	0.001

### Knockdown of BPTF by shRNA inhibited NSCLC cell growth *in vitro* and *in vivo*

**Figure 7:**
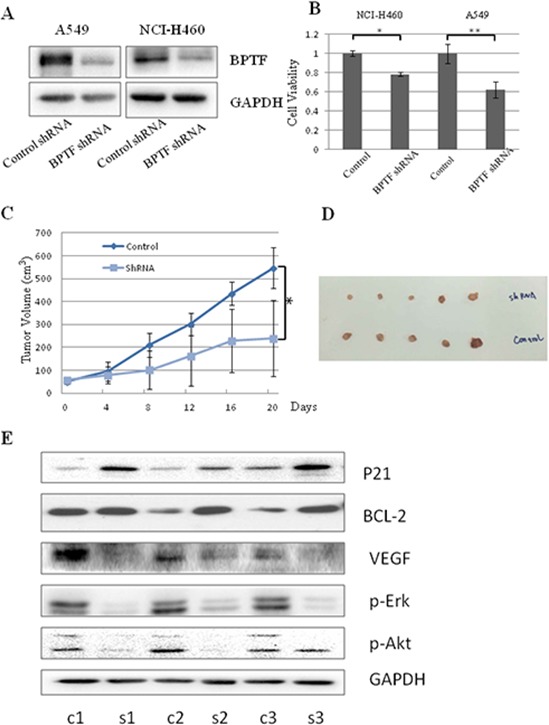
Knockdown of BPTF by shRNA inhibited lung cancer cell growth *in vitro* and *in vivo* **A.** The expression of BPTF in A549 and NCI-H460 cell lines were analyzed by Western blot after transfected with non-specific control shRNA or BPTF shRNA. **B.** Cell viability of A549 and NCI-H460 cells was measured by MTT assay. The mean and SD value got from 3 independent experiments are marked (*P < 0.05; **P < 0.01). **C.** Tumor growth curves in xenografts of A549 cells for each group (*n* = 5). Dots represent the mean, while bars indicate the SD. (*P < 0.05). **D.** Tumor graft from the 2 different groups treated with BPTF shRNA and Control shRNA respectively. **E.** The expression of BPTF, VEGF, P-Erk, P-Akt, P21, BCL-2 and GAPDH were examined from 3 pairs of tumor xenografts in non-specific control shRNA and BPTF-specific shRNA-treated nude mice by Western blot. “C” means control shRNA, and “B”means “BPTF shRNA”.

The effect of BPTF on lung cancer growth was further proved by using BPTF shRNA *in vitro* and *in vivo*. First of all, cell viability was measured by MTT assay at 48 h after A549 and NCI-H460 cell lines were transfected with control shRNA and BPTF specific shRNA. The results showed BPTF expression were markedly knocked down by its specific shRNA (Figure 7A) and its silencing led to the inhibition of cell viability in A549 and H460 cell lines (Figure 7B). To determine the role of BPTF in the regulation of tumor growth in a lung cancer mouse model *in vivo*, A549 cells were injected subcutaneously into the armpit of nude mice. When the tumor grew to 5 × 5 cm (lenth × width) in size, the animals were classified into 2 groups with 5 nude mice each group. The BPTF shRNA and control shRNA were respectively injected into nude mice every 4 days. The tumor volume was measured and recorded every 4 days too. 24 days later, animals were sacrificed and tumors were isolated to be weighted and examined. Compared to the control shRNA treated group, the tumor volume and weight were significantly reduced in the group treated with the BPTF shRNA (Figure 7C, 7D). Next, we also examined the effect of BPTF shRNA on the expression of some key proteins in the tumor tissues by Western blot. Compared to the control shRNA-treated group, the expression of VEGF, p-Erk, and p-Akt was markedly attenuated in the group treated by BPTF shRNA, while the expression of P21 and BCL-2 was improved (Figure 7E). All these results demonstrated again the silencing of BPTF inhibited lung tumor growth *in vitro* and *in vivo*.

## DISCUSSION

As the largest unit of NURF, BPTF is involved in chromatin remodeling which is a key process for eukaryotic cell. It is reported that BPTF facilitates gene transcription and accelerates cell development [12, 17, 18]. As a chromosome-mediated transcription factor, BPTF simultaneously recognizes 2 different histone modifications. The PHD finger of BPTF can bind dimethylated and trimethylated H3K4 (H3K4me2/3). Its adjacent bromodomain interacts with H4 peptides acetylated at K12, K16, or K20 [22–24]. Its function as a transcription factor is closely related with tumorigenesis. Francisco X Real identified BPTF gene recurrently mutated in bladder cancer by the method of exome sequencing [20]. Varda Rotter detected 17q24.3 region in the BPTF locus and found BPTF was associated with lung premalignant and 27% of lung tumors exhibited gain of 17q24.3 [21]. Also, Lu's group reported that there was some relationship between BPTF and hepatocellular carcinoma [25]. Most importantly, Altaf A. Dar demonstrated the vital role in melanoma progression [26]. Similarly, we detected the expression of BPTF protein expression in 75 lung adenocarcinomas and found 71% of them were positive. These results indicate BPTF is a novel biomarker in lung cancers.

In this study, we also investigated the biological functions of BPTF on cell growth, apoptosis and cell cycle, and found that BPTF played a key role in promoting cell proliferation, inhibiting cell apoptosis and accelerating cell cycle. The result of colony formation was similar to the previous reports that BPTF knockdown led to a great reduction in colony formation in lung, bladder cancer and melenoma [20, 26]. Especially, we proved BPTF has marked effect on tumor growth *in vivo*. In addition, we also identified the underlying mechanisms of these biological functions *in vitro* and *in vivo*. We found BPTF significantly promoted cell proliferation mainly by regulating the MAPK and PI3K-AKT pathways, which was similar to the report in melanoma but different from the report that BPTF promoted cell development and differentiation through TGF-Smad pathway [17, 26]. It is well known that extracellular regulating kinase (ERK) pathway is markedly related with cell proliferation, transforming and differentiation [27]. It is worth recalling here that the upstream protein c-Raf in ERK pathway was influenced, and the proteins in this pathway were also reduced subsequently after BPTF was knocked down. In contrast, another key protein involved in carcinogenesis and development, p38, was up-regulated by BPTF knockdown. The phospho-p85, an upstream protein in PI3K-AKT pathway, was down-regulated when BPTF was knocked down, which resulted in a reduction of the downstream proteins in this pathway, such as phospho-PDK1, phospho-Akt, and phospho-GSK-3β.

It is known that Raf is activated by RAS, which is stimulated by the active receptor tyrosine kinases (RTKs) in MAPK pathway[27]. Also, in PI3K-AKT pathway, autophosphorylation of ligand-activated receptor tyrosine kinases (RTKs) causes recruitment of inactive heterodimeric class IA phosphatidylinositol 3-kinases (PI3Ks) through the interaction of phosphotyrosine residues on the receptor and SRC-homology 2 (SH2) domains on the PI3K p85 regulatory subunit. These SH2–phosphotyrosine interactions bring PI3K in close proximity to its substrate at the plasma membrane and relieve the inhibitory action of p85 on the p110 catalytic subunit, which is then free to convert PtdIns(4,5)P2 (PIP2) into PtdIns(3,4,5)P3 (PIP3). Alternatively, binding of PI3K to activated RAS can also stabilize its membrane localization and activate the catalytic domain [28, 29]. Therefore, both of the pathways are activated by RTKs and RAS. In this study, knockdown of BPTF decreased the expression level of c-RAF and p-P85, the respective upstream proteins in the PI3K-AKT and MAPK pathways. Therefore, we speculate BPTF might promote the NSCLC proliferation by affecting the activity of RAS or RTKs. Furthermore, we checked VEGF expression when BPTF was knockdown by transecting BPTF shRNA *in vivo*. The result showed the VEGF were obviously down-regulated after BPTF was knocked down. Therefore, we speculated BPTF as a transcriptional factor regulated the expression of VEGF. When VEGF binds to VEGFR, the latter occurs autophosphorylation and then activates the downstream molecular such as Ras and P85 in the pathways of MAPK and PI3K.

We also discovered that BPTF restrained NSCLC apoptosis by regulating the activation of caspase-8, caspase-7 and PARP1 proteins. Apoptosis pathway includes mitochondrial regulation, direct signal transduction, and endoplasmic pathways [30]. We analyzed the mitochondrial regulation pathway by detecting related proteins, but unfortunately, we found that knockdown of BPTF up-regulated BCL-2 and down-regulated Apaf-1 and cleaved caspase-9 in the NCI-H460 cell line. The results indicated NSCLC apoptosis induced by BPTF may not be realized through the mitochondrial pathway. Considering that knockdown of BPTF increased the expression of cleaved caspase-8, it is possible that BPTF restrained apoptosis via the death receptor pathway, which needs to be further proved.

Furthermore, we detected BPTF-mediated effect on cell cycle in lung cancer A549 and H322 cells. Although the efficiency of BPTF knockdown by siRNAs was different, the cell cycle was obviously blocked from G1 to S phrase. We also detected the cell cycle related proteins by Western blot to investigate the deeper molecular mechanisms. As a chief external signal sensor, CyclinD plays a major role in the middle of G1 phase. Growth signals passed by Ras-Raf-MEK-MAPKs signal transduction pathway could finally enter nuclei and activate CyclinD1. By combining with CDK4/6, CyclinD1 is further activated to accelerate cell cycle by phosphorylating Rb and inactivating the latter. By contrast, when the level of phosophorylated Rb is attenuated, through binding to the transcriptional factor E2F, it could inhibit the transcriptional activity of E2F, thus suppressing cell cycle from G1 to S phase. Instead, if Rb is phosphorylated, E2F promotes transcription of many genes, thus starting a cell to enter S phase [31–34]. The stability of Cyclin D itself is controlled by glycogen synthesis protein kinase -3beta, GSK-3β. Under the condition of lacking of growth signals, GSK-3β is able to be activated to phosphorylate Cyclin D and promote its degration. As a result, cell cycle is blocked. In this study, we found that the knockdown of BPTF could down-regulate the levels of phosphor-AKT and phospho-GSK-3β, and then reduced the expression of Cyclin D1 and phospho-Rb, and further inhibited cell cycle. As CDK kinase inhibitor, p18 and p21 were up-regulated [35–37]. We can also see P21 was up-regulated when BPTF was knocked down by shRNA *in vivo*. All the data well explained the molecular mechanism of the blocking from G1 to S phase in cell cycle caused by knockdown of BPTF. Moreover, we tested phospho-cdc2 involved in the checkpoints from G2 to M and found that it was decreased in BPTF knockdown groups, indicating that BPTF might inhibit cell cycle from G2 to M phrase too [38]. Taken together, we showed that BPTF was a key factor to promote cell cycle in NSCLC.

We also found BPTF was highly expressed in NSCLC cell lines and tumor tissues compared to the normal cell or adjacent tissues. However, in our study, we found the high expression of BPTF at mRNA level but low expression at protein level in H1299 cells. This probably resulted from the post-translational modification of BPTF. The exact regulatory mechanism for this possible post-translational modification deserves a further study.

To see whether BPTF played a vital role in human clinical samples, we analyzed the data from 75 cases with lung adenocarcinoma. We found the high expression of BPTF was associated with N (lymph node metastasis) and clinical staging factors. It was reported that VEGF was closely related with lymph node metastasis and tumor angiogenesis. We found VEGF was decreased with the knockdown of BPTF *in vivo*. Therefore, it is possible that the association of BPTF with N ( lymph node metastasis) is through its effect on VEGF. Lifetime of lung adenocarcinoma was associated with clinical stage and BPTF expression. We also have demonstrated that the overall survival of patients with high BPTF is shorter than that with low BPTF expression according to Kaplan–Meier analysis. Therefore, BPTF is not only a key factor *in vitro* or in nude mice, but also a crucial one in human tissues.

Collectively, our results demonstrate that BPTF plays an essential role in NSCLC cell survival, and it may be a potential therapeutic target for NSCLC.

## MATERIALS AND METHODS

### Cell lines and cell culture

Human normal lung fibroblast (HLF-1), human NSCLC cell lines (A549, NCI-H460, H322, and H1299) were purchased from the American Type Culture Collection (ATCC, Manassas, VA). HLF-1 was cultured in F12K medium. A549 was cultured in DMEM/F12 medium. H322, NCI-H460 and H1299 were cultured in RPMI-1640 medium. All the mediums were supplemented with 10% fetal bovine serum (Biological Industries, Israel) and produced by Invitrogen Company (Carlsbad, CA). Cells were maintained in a humidified atmosphere and 5% CO_2_ at 37°C.

### Western blot analysis

Protein samples with proteinase and phosphatase inhibitors (Thermofisher, USA.) were mixed with SDS sample loading buffer and resolved by PAGE. 30–40 μg total protein was transferred to 0.22 μm or 0.45 μm PVDF (Millipore, Germany) overnight at 20–30 V limits. Membranes were blotted with antibodies of BPTF (Abcam, USA) at 1:2000, β-actin ( proteintech, USA) at 1:5000, GAPDH (Proteintech, USA) at 1:5000, phospho-PI3Kp85(Tyr458)antibody (CST, USA) at 1:1000, PI3K p110-γ (CST, USA) at 1:1000, Phospho-p38 (Thr180/Tyr182) (3D7) (CST, USA) at 1:1000, p38 at 1:1000, Cleaved Caspase-7 (Asp198) at 1:1000, BCL-2 (Proteintech, USA) at 1:1000, Cleaved PARP (Asp214) (D64E10) (CST, USA) at 1:1000, Phospho-cdc2 (Tyr15) (10A11) (CST, USA) at 1:1000, Phospho-Rb (Ser795) (CST, USA) at 1:1000, Phospho-p53(Ser15) (16G8) (CST, USA) at 1:1000, and Phospho-Akt (Thr308) (CST, USA) at 1:1000, Phospho-Erk1/2 Pathway Antibody Sampler Kits (CST, USA) at 1:1000, adding cell cycle regulation antibody sampler kit (CST, USA) at 1:1000 and Cell Cycle/Checkpoint Antibody Sampler Kit at 1:1000 overnight at 4°C, then anti-rabbit or mouse HRP (Proteintech, USA) at 1:5000–1:10000 dilutions for about 2 hrs at RT in TBST. At last, protein brands were detected by ECL (Advansta, USA) according to manufacturer's procedures.

### RT-PCR

Total RNA was extracted and reversely transcribed into cDNA according to the instructions of RNAiso Plus kit and PrimeScript ^™^ RT reagent Kit with gDNA Eraser (TaKaRa, China). The cDNA was amplified by PCR following the protocol of TaKaRa Taq ^™^ Hot Start Version (TaKaRa, China). The sequence of forward primer of BPTF was 5′-AATCGGAGAAGTCCAACGGG-3′; the sequence of reward primer of BPTF was 5′-TTGCCCTATGTGATGCCCAG-3′. They were produced by life Technologies Company (Invitrogen, Carlsbad, USA).

### Human lung adenocarcinoma tissue microarray

The human lung adenocarcinoma tissue microarray was purchased from Shanghai Outdo Biotech (China) consisting 75 cases with lung adenocarcinoma and their adjacent non-malignant lung tissues. These cases had got nothing anticancer therapies before tumor resection. We have the detailed clinical and pathological information, including age, sex, chief complaints, TNM stage, and overall survival (OS) duration and so on.

### Transient transfection of siRNA

siRNAs were synthesized by Shanghai Genepharma Company (China). Non specific siRNA and three BPTF-siRNAs were designed. Two of the three BPTF si-RNAs were effective, and for the knockdown efficiency, BPTF siRNA-1 was not as validated as BPTF si-RNA-2. Their sequences were: non specific siRNA: 5′-UUCUCCGAA CGU GUCACGUTT-3′ and 5′-ACGUGACACGUUCGGAGAATT-3′; BPTF-siRNA-1 (BPTF-homo-1550): 5′-GGUCCAACUUGCAGAAUUATT-3′ and 5′-UAAUUC UGCAAGUUGGAC CTT-3′; BPTF-siRNA-2 (BPTF-homo-6959): 5′-GACCCA AACAACUGUUUCATT-3′ and 5′-UGAAACAGUUGUUUGGGUCTT-3′. The procedure of transfection was as followed. First, cells were seeded into each well of a six-well tissue culture plate and estimated at 60–80% cell confluency the next day. At the same time, put the well mixed reagent with RNAiMax (Invitrogen, Carlsbad, USA) and siRNA into still suspending cells following the instruction of RNAiMax. The next day, when cells adhered onto culture plates, we change the culture medium with 10% FBS but without antibiotics. After 48 or 72 hrs, cells were collected and the related experiments were done.

### Transient transfection of shRNA

ShRNAs were purchased from Gene Copoeia Company(U.S.A). Four shRNAs were designed, and one of them was the most effective (Catalog No.: HSH005096-LVRH1MP and CSHCTR001–1-LVRH1MP). According to the lipofectamine 3000 protocol (Invitrogen, U.S.A), control shRNA and BPTF specific shRNA were transfected into A549 and NCI-H460 cell lines. After 48 h, MTT assay was used to measure cell viability.

### Cell proliferation and colony formation assay

Cell proliferation was estimated by ways of MTT (Promega) array according to the manufacturer's protocol. Cell viability was measured after 24, 48 and 72 hrs. At last, it was identified that the difference was the most obvious when cells were transfected for 72 hrs. To do colony formation assay, 1000 cells were seeded into each well of six-well plates, at the same time the cells were transfected with siRNAs. The next day, changed new culture medium with antibiotic, cells was put into the incubator again. Cells were cultured for about 12 days without any disturbing, and colonies were stained with crystal violet for 10–30 minutes at RT. At last colonies were counted by eyes.

### Apoptosis and cell cycle assay

Cells (A549 and NCI-H460) transfected with si-RNA were analyzed by flow cytometry to detect apoptosis. After 72 h, the transfected cells were stained with Annexin V-FITC and PI (propidium iodide) according to the instruction of Keygen Annexin V-EGFP Apoptosis Detection Kit (China) and were placed on ice for 5–15 minutes in the dark. Then samples were examined by flow cytometry (BD FACScalibur).

Cell cycle analysis was carried out by PI staining (Sigma, USA). The transfected cells for 72 h were harvested and fixed in 70% cold ethanol overnight (not more than 1 week). After removing the disturbed RNA by adding RNase (Thermofisher, USA) at 37°C for 30 minutes, cells were stained with PI on ice for 10–20 minutes. At last, samples were performed by flow cytometry (BD FACScalibur).

### Immunohistochemistry

The tissue microarray slides were deparaffinized in xylene and rehydrated through graded alcohol. Antigen retrieval was performed by incubating samples with Target Retrieval Solution (pH 9; DakoCytomation) for 15 minutes using a pressure cooker. Then the slides were immersed in methanol containing 3% hydrogen peroxide for 20 minutes to block endogenous peroxidase activity. After preincubation in 2.5% blocking serum to reduce nonspecific binding, the sections were incubated overnight using primary antibody, anti-BPTF (Bethyl, 1:50 dilution), in a humiditified container at 4°C. The tissue microarray slides were processed with horseradish peroxidase immunochemistry according to the manufacturer's recommendations (DakoCytomation, CA). As a negative control, the staining procedure was performed with the primary antibody replaced by a normal rabbit IgG. Staining intensity was graded as: absent staining as “-”, weak as “+”, moderate as “++”, strong as “+++”. Because each tissue on the tissue microarray slides was quite small, we could not determine the grade of percentage of stained cells. Staining tissues with “++”, “+++” were determined as “high expression of BPTF”, but the other tissues with “-” and “+” were judged as “low expression of BPTF”. The result was identified by two senior pathologists.

### Statistical analysis

Pearson chi-square test was used to analyze the relationship among BPTF and gender, age, T, N, clinical stage. Cox regression model was performed to analyze factors related to survive. We adopted stepwise regression model: Forward: LR, selected variables (*P* ≤ 0.05); Eliminating variables (*P* > 0.05). Univariate analysis was used to assess the relation of gender, age, T, N, clinical stage and BPTF expression to oversurvival of lung adenocarcinoma. And multivariate analysis was performed to further show the influent factors to overall survival of lung adenocarcinoma. At last, Kaplan–Meier analysis was performed to assess the association between the overall survival and BPTF expression. *P* value < 0.05 was considered statistically significant in all cases.

### Animal experiments

Animal experiments were carried out according to the National Institue of Health Guide for the Care and Use of Laboratory Animal under the approval of APF laboratory Animal Center at Dalian Medical University. A549 cells (5 × 10^6^) were planted subcutaneously under the left arms of nude mice. After about 14 days, tumors grow to 5 mm × 5 mm (length × width), and the nude mice were classified into 2 groups. There was no difference between the 2 groups on tumor volumes and the size of nude mice. Group 1 was set as control shRNA, and Group 2 was BPTF shRNA. According to lipofectemine 3000 protocol, we transfected shRNA plasmids into tumors. 25 μg shRNA in 100 μl liposome was injected per 4 days for 20 days. The tumor volume was calculated in terms of “Volume = (width^2^ × length)/2” using digital calipers. In the end, the nude mice were sacrificed for further checking the expression of related proteins by Western blot.
